# The Effect of Parental Socioeconomic Class on Children’s Body Mass Indices

**DOI:** 10.4274/Jcrpe.898

**Published:** 2013-05-30

**Authors:** İbrahim Al Alwan, Areej Al Fattani, Nick Longford

**Affiliations:** 1 King Saud bin Abdulaziz University for Health Sciences, Riyadh, Saudi Arabia; 2 King Abdulaziz Medical City, Riyadh, Saudi Arabia; 3 Ministry of Health, Epidemiology and Biostatistics Department, Riyadh, Saudi Arabia; 4 SNTL Statistical Research and Consulting, Barcelona, Spain

**Keywords:** children, overweight, obesity, socioeconomic, education

## Abstract

**Objective:** To assess the effect of education and economic status of parents on obesity in children.

**Methods:** A cross-sectional survey was conducted in 2006 among school children in Riyadh, Saudi Arabia. A representative sample of 1243 (542 male and 701 female) children aged 6-16 years were contacted using multistage cluster sampling strategy. Social and demographic variables were collected using questionnaires completed by parents. Height and weight of the children were recorded by a trained team.

**Results:** The mean body mass index for all children was 19.8±5.4. The prevalence rates of overweight and obesity were 21.1% and 12.7%, respectively. Overweight and obesity were more prevalent in males than in females. By multivariate analysis, children were more likely to be overweight if they were male (OR=0.6, p<0.01), 12 years of age (OR=3.79, p<0.01, compared to age 6 years), and if their families had higher income (OR=3.12, p<0.01, compared to families with low income). Being male (OR=0.545, p <0.01), aged 12 years (OR=3.9, p=0.005, compared to the age of 6), and having a mother who is more educated were determined to be significant risk factors for obesity in children. Mothers educated at university level were found to have a three-fold higher risk of having obese children(OR=3.4, p<0.01, compared to mothers with lower education levels).

**Conclusions:** Overweight and obesity among Saudi children is associated with educated mothers and higher family income. This finding calls for introducing interventions in health education for both children and parents.

**Conflict of interest:**None declared.

## INTRODUCTION

Overweight and obesity in childhood and adolescence are among the most serious health problems in developed countries (1,2). The occurrence of overweight and obesity has been rising over the 20th century and recently has risen even more dramatically (3). In 2010, the World Health Organization (WHO) estimated that approximately 43 million children under five years of age are either overweight or obese (4). Obesity rates tend to continue into adulthood and are combined with early weight-related health problems (5). Cardiovascular disease, diabetes, hypertension, and hyperlipidemia are common long-term complications for obese children (6). Therefore, obesity not only affects the child’s quality of life but also increases the burden on health care services because of chronic complications (7). There are several factors that play a role in obesity: genetic factors, race, and some clinical conditions, such as hypothyroidism. However, the environment has the biggest impact on the rapidly increasing obesity prevalence in a short time frame (8). Behaviors such as increased use of motorized transport, decreased physical activity, and increased consumption of high-energy foods and drinks are now common in developing countries following the improvement in economic conditions in Eastern Europe and the Middle East (9). Socioeconomic status (SES) and education level act as confounders that contribute indirectly to childhood overweight and obesity. In developed countries such as the United States of America (USA) and the United Kingdom (UK), obesity has been associated with lower SES and a lower parental education level (10,11). Low SES may be associated with a variety of factors in developed countries, including health insurance, local food stores and the extent to which they carry healthy foods, the price of food, the tendency to watch television and participate in other sedentary activities, and access to health clubs (12). In contrast, obesity in developing countries has been associated with higher SES and urbanization. Studies in Brazil, China, and India support this association (3,13). In Middle Eastern and Gulf countries, the prevalence of obesity varies widely. Kuwait has the highest recorded prevalence of obesity in preschool children (9%) and the highest prevalence of overweight in adolescents, reaching 30% (14,15). Higher overweight and obesity statistics have been recorded in urban areas and more affluent communities in Jordan, Iran, and Egypt (3,16,17). In Saudi Arabia, data from a national survey conducted in 1998 show that approximately 27.4% of Saudi children aged 1-18 years were overweight and 10.4% were obese. The study also shows variations of prevalence among the regions of Saudi Arabia (18). Studies in the Eastern region of the kingdom present higher occurrences of overweight and obesity compared to other regions where higher economical status and different lifestyles are prevalent (19,20). Recently, national data from the Saudi Census in 2005 have been analyzed by El Mouzan et al (21). They concluded that in a sample of children aged 5-18 years, the prevalence rates of overweight and obesity were 23.1% and 9.3%, respectively (21).

The aim of this study was to provide data about obesity and overweight among children and adolescents in Riyadh city and to assess the effect of education and economic status of the parents as risk factors. Such information may be valuable in developing programs and policies to combat the epidemic of obesity among school children in Saudi Arabia.

## METHODS

**Study Design and Population**

A cross-sectional survey was conducted in Riyadh from January to June 2006 among school children. The data analyzed in this project were part of the Riyadh Puberty Study (22). The sample was drawn by a multistage cluster strategy. Riyadh was divided into four regions represented by the four directions of the compass. From each region, a private and a public school unit for boys and for girls were selected randomly to represent different economic status of the Riyadh population in the sample. These data were provided by the Ministry of Education and Ministry of Planning. From each school, more than ten students from each grade were invited to participate. Students were included in the study if they were 6 to 16 years of age, did not have any chronic diseases, and were not taking any long-term medication. A total of 1243 students (542 male and 701 female) provided complete data for the analysis.

**Ethical Approval**

The study protocol was approved by the Research and Ethics committee at the King Abdullah International Medical Research Center in Riyadh. A parent’s informed consent and the agreement of the child for physical examination and blood sample drawing were collected with the questionnaires.

**Data Collection**

A pilot study was previously conducted on a school belonging to the National Guard Housing to test the efficacy of the questionnaire. Two weeks before field visits to schools, each student in the sample received an information leaflet about the study along with a questionnaire to be answered by parents or guardians. The questionnaire included questions about demographic data, parental education level and occupation, family income, and medical history of the child. Anthropometric data were collected by a trained team of pediatric residents, nurses, and research coordinators in the school’s clinic or a specifically designated area. Height was measured using a wall-mounted stadiometer, with the children not wearing shoes and their shoulders in a relaxed position and their arms hanging freely. The measurements were recorded to the nearest 0.1 cm. Weight was measured to the nearest 0.1 kg with a beam-balance scale, which was re-calibrated for every new subject. Subjects were weighed barefoot and wearing minimal clothing. Obesity and overweight were defined using the WHO 2007 growth standards (23). Cutoff values were applied to our data after calculating the nutritional indices by the NutChildren program in the Epi Info 3.5.1 statistical software.

**Statistical Analysis**

Mean, standard deviation, frequency, and percentage of all factors were used to report the data. A chi-square test and t-test were used wherever appropriate. Multivariate analysis using binary logistic regression was conducted to determine factors associated with overweight and obesity. In the logistic models, overweight and obesity (each categorized as a dichotomous variable) served as the dependent outcome variables. All independent categorical covariates were tested in the models estimated using the backward conditional method. Data were analyzed using SPSS version 17. For each variable in the equation, the odds ratio [exp (B)], confidence intervals for exp (B), and the significance were obtained. A p-value of less than 0.05 was considered to be statistically significant. 

## RESULTS

**Participants Characteristics**

After excluding 31 children who were assessed as ill by medical history and a physical examination, the study included 1212 children (42.2% boys and 57.8% girls) between 6 and 16 years of age. The mean age was 11.18 years for girls and 10.80 years for boys. About 27.5% of the fathers and 22.5% of the mothers had completed their university education. Most fathers and mothers (>60%) were government employees, and only 1.6% were unemployed. Approximately 12.3% of the families had low income (less than 3000 SRs/month), 65.2% had an average income (between 3000 and 19999 SRs/month with a mean of 11000 SRs/month), and 9.6% of families had a high income (20000 SRs/month or higher).

**Anthropometric Measurements**

**Figure 1:**
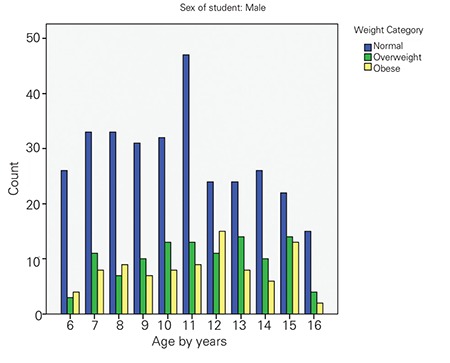
The prevalence of normal, overweight and obese boys byage group

**Figure 2:**
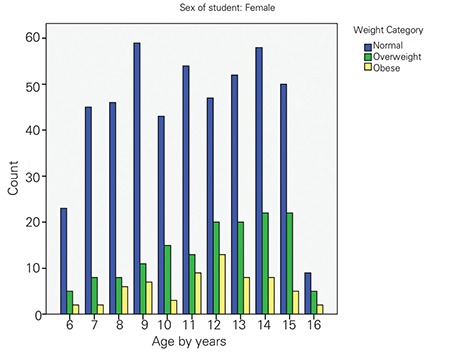
The prevalence of normal, overweight and obese girls byage group

The mean height of the participants was 143.6±16.5 cm and 144.3±14.7 cm in males and females, respectively. The mean weight of male and female participants was 43.5±20.6 kg and 42.6±17.2 kg, respectively. Mean body mass index (BMI) of all participants was 19.8±5.4 kg/m2 (20.2 for boys and 19.7 for girls). Boys had the same average height as girls but were heavier on average. The prevalence of overweight and obesity was 21.1% (21.5% for boys and 21.3% for girls) and 12.7% (17.4% for boys and 9.3% for girls), respectively. The counts of obese and overweight children for every age group of girls and boys are shown in Figures 1 and 2. The highest prevalence of overweight for boys was at 13 and 15 years of age and at 14 and 15 years of age for girls. The highest prevalence of obesity for boys and girls was at 12 years of age. Table 1 shows the distribution of all participants according to the studied variables and the univariate analysis against overweight and obesity outcomes.

Using a multivariate logistic regression, the significant covariates were assessed against overweight as a dichotomous outcome (Table 2). Boys are at twice the risk of being overweight as girls (OR=0.6, p<0.01 with 95% CI: 0.51-0.86). Compared to 6-year-old children, children 12 years of age are about three times more likely to be overweight (OR=3.53, p<0.01 with 95% CI: 1.73-7.11), and 13- and 15-year-old children are approximately twice as likely to be overweight. The risk seems to increase with age without an obvious trend. Overweight also increases with increased family household income. Compared to low-income families, families with an average income show twice the risk of having overweight children. High-income families are at about a three-fold higher risk for having overweight children (OR=3.38, p<0.01 with 95% CI: 1.90-6.02).

Table 3 shows the significant covariates associated with obesity in the multivariate analysis. Boys have a significantly higher risk than girls (OR=0.50, p-value<0.01 with 95% CI: 0.35-0.71). Obesity seems to increase with an increased level of the mother’s education without any obvious trend. Compared to illiterate mothers, mothers educated to an intermediate level have about a four-fold higher risk of having obese children (OR=4.0 p<0.01 with 95% CI: 1.68-9.51), mothers educated to the high school level have about a two-fold higher risk (OR=2.7, p<0.05 with 95% CI: 1.20-5.33) and mothers educated to the university level have about a three-fold higher risk (OR=3.7, p<0.01 with 95% CI: 1.62-8.48). 

## DISCUSSION

This study is the first to assess the prevalence of obesity and overweight with a representative sample in Riyadh city and simultaneously evaluate the socioeconomic risk factors. In the studied sample, which included 6-16 years old participants, the prevalence of overweight was 21.1%. This figure is lower than in Kuwait, where about 30% of adolescents are overweight (15), but higher than in Jordan (19.4%). Notably, this survey covers only Riyadh city, not the whole country. Compared to national multiregional studies (21), this prevalence seems to be high. A higher prevalence of overweight in large cities compared to rural areas and villages was also observed in El Hazmi’s comparative study (18). In our results, the prevalence of obesity was 12.7% (17.4% for boys and 9.3% for girls). A higher prevalence of obesity among boys is consistent with the El Mouzan et al’s study in 2010 (21) and also with the WHO’s recent review of childhood obesity in the Middle East, which corroborates evidence showing the increasing occurrence of obesity among boys compared to girls (24).

Our results indicate that prevalence of overweight increases with age by significant probability ratios. This finding might be more obvious if our study had included adolescents over 16 years old. This finding is consistent with a three-year longitudinal Australian study which found that in a group of 5-6-year-old children, prevalence of overweight increased from 19% to 28% over three years (25). Furthermore, many studies in Saudi Arabia and Gulf countries confirm that prevalence of overweight increases with age (24,26).

Our data show that the risk of becoming overweight among children tends to be greater with higher family income. The association between obesity and high SES has been observed also in low- to middle-income countries (3,16,17). Two Saudi studies support this association. Alam (27) evaluated the prevalence of obesity among schoolgirls in a high-class district in Riyadh and found an obesity occurrence of 14.9%. Most girls had a sedentary lifestyle, and 95% lived in villas or big houses, indicating middle to high SES in Saudi culture. In the Eastern region, Amin et al (19) studied obesity and overweight in schoolboys aged 10-14 years; 9.7% of them were obese and 14.2% were overweight. There was a significant correlation between obesity and higher SES. It is believed that high-income families can afford several meals per day, eat out in restaurants, have food delivered to the home, and have more frequent snacks and other foods with high caloric content. In contrast, low-income families tend to save money by eating economic meals at home one or two times daily (24).

The multivariate regression of obesity as an outcome shows that obesity is affected by sex and mother’s education level. This finding is in contrast to what has been recorded in the USA (10), the UK (28) and Australia (29) regarding this association. In those studies, obesity was more common among parents with a low education level, which is associated with low income. Based on our data, a higher education level of the mother would not be explained by a higher family income. The mother’s education remains significant even after adjusting for other factors, such as income and the mother’s employment status and occupation. From the literature search, we noticed a relationship between working mothers and having obese children (30,31). This was explained by the mother’s long absences from home, which expose their children to unhealthy dietary habits of snacks and skipped meals (32). With that assumption, it seems that highly educated mothers in Saudi Arabia spend more time away from their children either in college, studying, at work, or in social activities. Consequently, most of those mothers depend on foreign caregivers or housemaids to care for their children. A study in Saudi Arabia has shown that 89% of Saudi families have at least one servant, of which 79% are of non-Arabic origin (33). This may reduce the chances for direct supervision and control by the mother, resulting in an increased risk of childhood obesity, especially if junk food is easily accessible for children and adolescents.

This study has some limitations. Firstly, the study design is cross-sectional. A longitudinal study would be best to assess causal relationships. Secondly, the study is limited to one city and its urban population.

In conclusion, this study demonstrates that overweight and obesity are serious public health problems among children in Saudi Arabia. In Riyadh, boys are affected more than girls, children of families with a higher income are more likely to be overweight, and children of educated mothers are more likely to be obese. An urgent systemic and comprehensive intervention is needed in the fields of health and nutrition, including educational programs for parents and children in schools. Further studies are needed to assess the effect of other contributing factors to prevalence of overweight and obesity.

## Figures and Tables

**Table 1:**
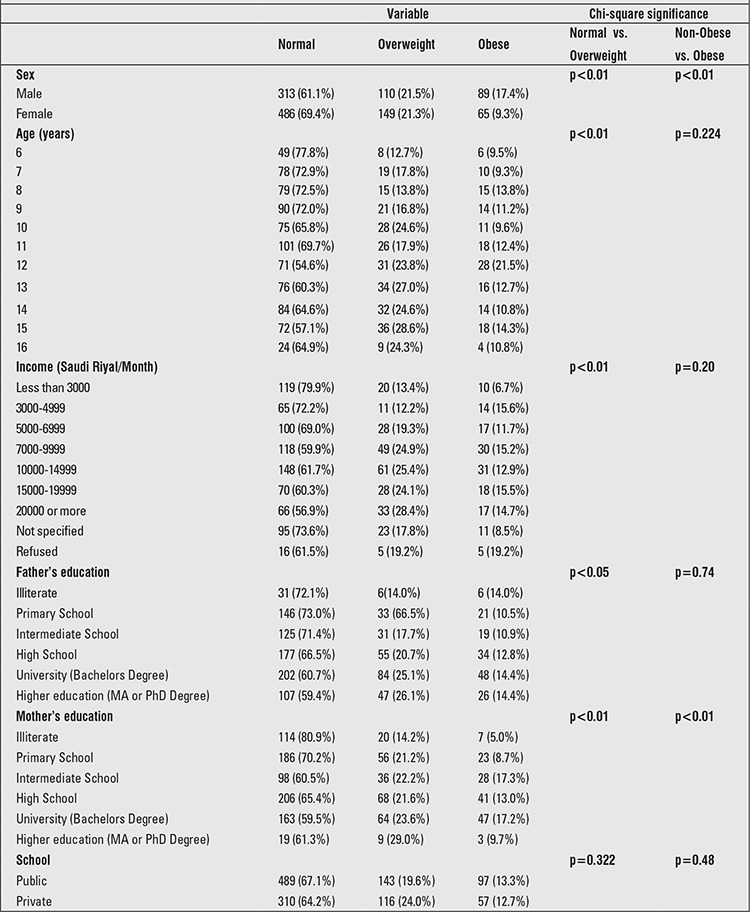
Distribution of the participants by studied risk factors and their cross-tabs in relation to overweight and obese outcomes

**Table 2:**
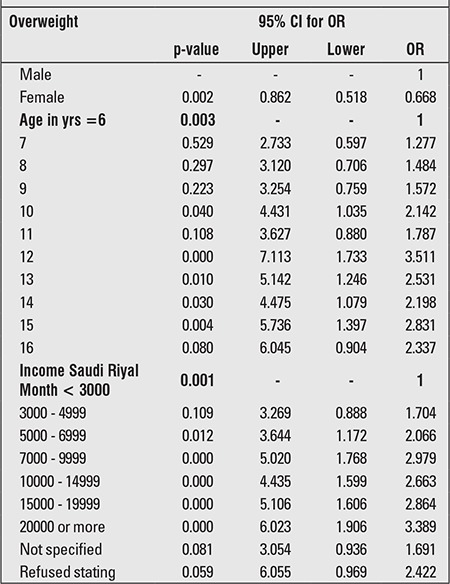
Multivariate analysis of overweight and variable coefficients

**Table 3:**
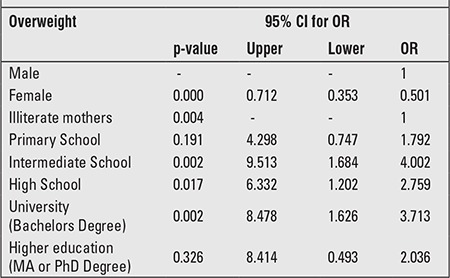
Multivariate analysis of obesity and variable coefficients
